# Appearance of CNS histoplasmosis on ^18^F-FDG PET/CT with MRI correlation

**DOI:** 10.1259/bjrcr.20150443

**Published:** 2016-07-28

**Authors:** William Makis, Rajan Rakheja, Stephan Probst

**Affiliations:** ^1^Department of Diagnostic Imaging, Cross Cancer Institute, Edmonton, AB, Canada; ^2^Department of Nuclear Medicine, Jewish General Hospital, Montreal, QC, Canada

## Abstract

Disseminated histoplasmosis is an opportunistic infection encountered in immunocompromised patients such as those with human immunodeficiency virus infection/acquired immune deficiency syndrome. Involvement of the central nervous system (CNS) can occur in 5–20% of cases of disseminated histoplasmosis, and CNS histoplasmosis can be very difficult to diagnose *via* conventional imaging modalities such as CT or MRI. The role of ^18^F-fludeoxyglucose positron emission tomography/CT scan in the diagnosis of CNS histoplasmosis has not been established. A 66-year-old female presented with dizziness and unsteady gait and was diagnosed with human immunodeficiency virus infection and CNS histoplasmosis. In this report, we present the MRI and ^18^F-fludeoxyglucose positron emission tomography/CT image findings.

## Case report

**Figure 1.**
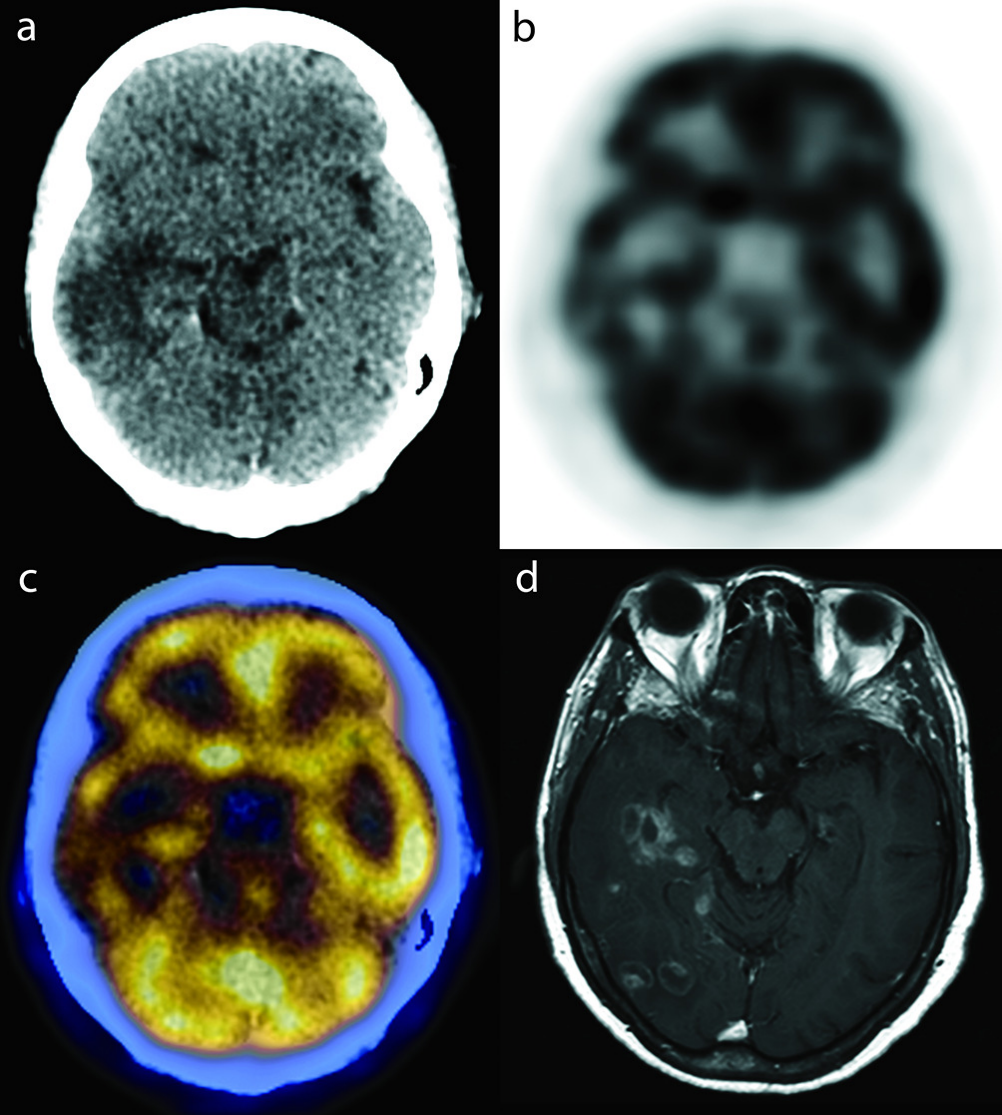
(a) Transaxial CT, (b) PET, (c) PET/CT fusion and (d) MRI *T*_1_ weighted post-gadolinium images. The intensely enhancing cerebral lesions seen on MRI appear hypometabolic on the PET/CT images when compared with normal grey matter. PET, positron emission tomography.

**Figure 2.**
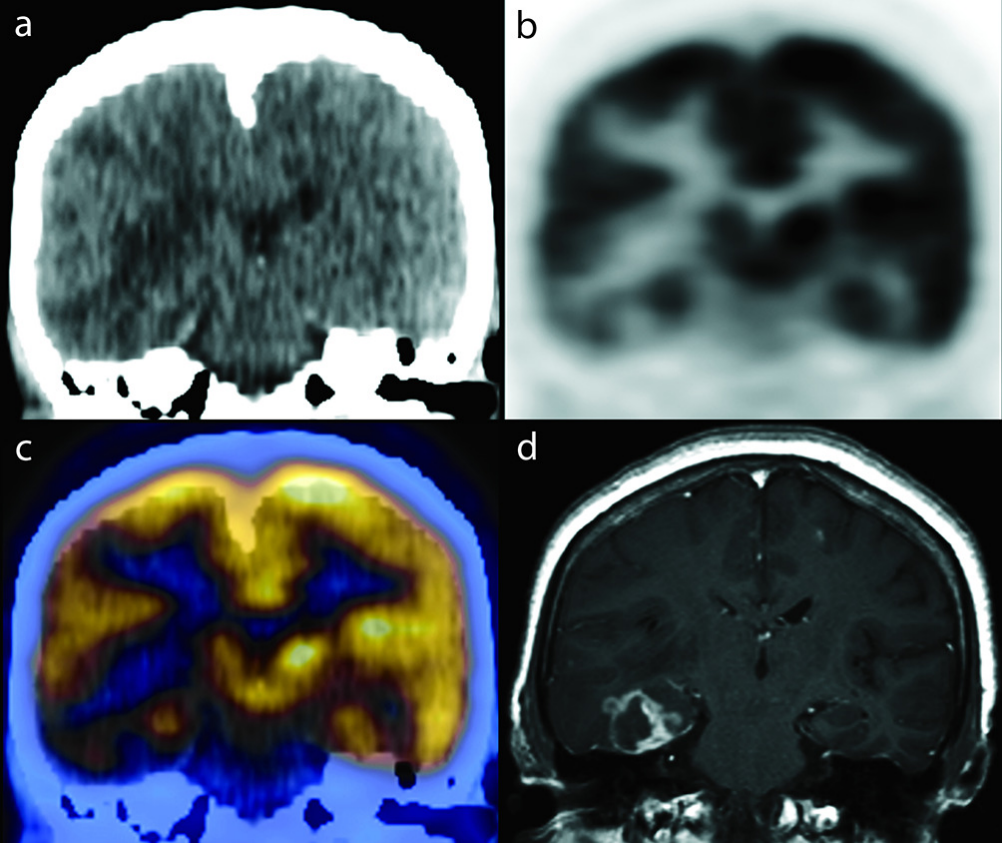
(a) Coronal CT, (b) positron emission tomography, (c) positron emission tomography/CT fusion and (d) MRI *T*_1_ weighted post-gadolinium images. The largest single lesion in the right temporal lobe measured 2.4 × 1.9 cm in the coronal plane.

**Figure 3.**
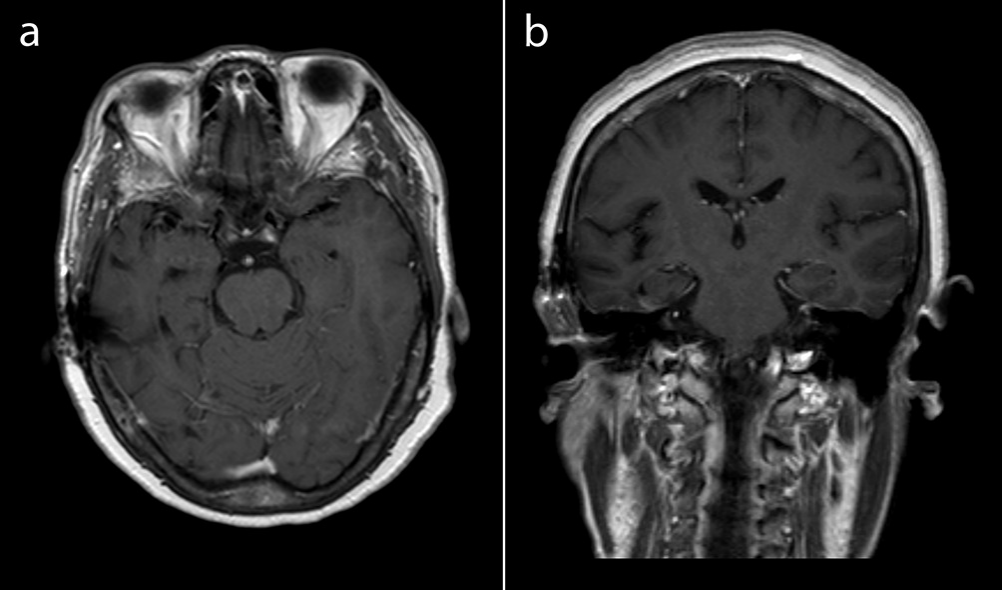
A follow-up MRI performed 2 years later with (a) transaxial and (b) coronal *T*_1_ weighted post-gadolinium images showed complete resolution of the right temporal lobe *Histoplasma capsulatum* lesions.

A 66-year-old female, who was previously well, presented with a 2-week history of night sweats, new onset headache, dizziness, intermittent double vision and unsteady gait. On physical examination, she walked with a left-sided hemiplegic droop, leaning towards the left side. She was initially referred for an MRI which, on *T*_1_ weighted post-gadolinium images, showed several lesions in the right temporal lobe, the largest measuring up to 2.4 × 1.9 cm with intense irregular ring-like enhancement (Figures 1d and 2d). The lesions were associated with *T*_2_ hyperintensity and the differential diagnoses of the MRI findings included high-grade astrocytoma, lymphoma, metastases and infection, and the patient was referred for an ^18^F-fludeoxyglucose (FDG) positron emission tomography (PET)/CT scan (Discovery ST, GE Healthcare, Waukesha, WI) to assess for possible malignancy. She was injected with 12 mCi of ^18^F-FDG and imaged approximately 75 min after the injection. The PET/CT scan failed to show extracerebral ^18^F-FDG-avid lesions, and the intensely enhancing cerebral lesions described on MRI were hypometabolic [maximum standardized uptake value (SUV_max_) 5.4] when compared with normal grey matter (SUV_max_ 10.1 for the basal ganglia and 7.6 for the sensorimotor cortex) (Figures 1a–c and 2a–c), which increased the likelihood of an infectious aetiology. Following the failure of a diagnostic/therapeutic trial of antimicrobials for toxoplasmosis, a brain biopsy and culture confirmed the presence of *Histoplasma capsulatum.* The patient was also diagnosed with human immunodeficiency virus (HIV) infection and was put on highly active antiretroviral therapy. Her central nervous system (CNS) histoplasmosis was treated with i.v. liposomal amphotericin B (at a dose of 3 mg kg^−1^ day^−1^) and her neurological symptoms gradually improved. A 2-year follow-up MRI showed complete resolution of the CNS histoplasmosis lesions (Figure 3).

## Discussion

*H. capsulatum* is a thermally dimorphic fungus and infects humans when it is inhaled into the lungs where it germinates into yeast. Disseminated histoplasmosis is an opportunistic infection encountered in immunocompromised patients, such as those with HIV/acquired immune deficiency syndrome (AIDS).^1,2^ While patients with chronic infection often present with pancytopenia and hepatosplenomegaly, patients with AIDS may present with much more serious signs and symptoms, including respiratory distress, shock and hepatic or renal failure.^3,4^

The CNS is involved in up to 20% of cases of disseminated histoplasmosis and can present as meningitis, multiple focal brain lesions or encephalitis.^5^ CNS histoplasmosis is very difficult to diagnose *via* conventional diagnostic imaging modalities such as CT and MRI, as the presenting brain lesion is often interpreted as a brain tumour or a focus of toxoplasmosis, as was the case with our patient.^6^ Diagnosis is usually confirmed by cerebrospinal fluid culture or biopsy, although these tests suffer from low sensitivities (as low as 20% for cerebrospinal fluid culture and 50% for biopsy).^7^ Treatment guidelines now recommend induction therapy with amphotericin B followed by itraconazole for life in patients with HIV/AIDS.^5^

Azizirad et al^8^ reported the only other known case of pathologically proven CNS histoplasmosis imaged with an ^18^F-FDG PET/CT scan, where they ascribed the increased glucose metabolism to the patient’s histoplasma lesion; however, careful review of the images showed only minimally increased uptake in the enhancing portion of the lesion when compared with the immediate soft tissue surroundings, and the average uptake of the entire lesion was well below the uptake in the normal grey matter, findings that are very similar to those in our case.^8^ Symptomatic CNS histoplasmosis is exceedingly rare and only a few cases have been reported in the imaging literature.^9,10^

Several studies have shown ^18^F-FDG PET/CT scans to be particularly useful in differentiating infections such as toxoplasmosis from malignant lesions such as lymphoma or metastases in HIV-positive patients. In a study of 25 patients by Lewitschnig et al,^11^ 10 of 11 patients with a diagnosis of toxoplasmosis were correctly diagnosed by PET/CT scan showing ^18^F-FDG uptake by the lesion to be less than that of normal brain cortex with a mean SUV_max_ of 3.5 (range 1.9–5.8), while malignant lesions such as CNS lymphoma showed ^18^F-FDG uptake greater than normal brain cortex with mean SUV_max_ of 18.8 (range 12.4–29.9). Interestingly, two patients with tuberculosis also showed low ^18^F-FDG uptake.^11^ Westwood et al^12^ showed that an ^18^F-FDG PET/CT scan was able to correctly identify lymphoma and hypometabolic toxoplasmosis in all of their 10 HIV-positive patients.

Our case showed that CNS histoplasma lesions were hypometabolic compared with normal grey matter, similar to ^18^F-FDG PET/CT results that have been seen with other CNS infections in HIV-positive patients such as toxoplasmosis or tuberculosis, suggesting that an ^18^F-FDG PET/CT scan may not be useful in the diagnosis of CNS histoplasma lesions, contrary to what has been reported in the literature thus far.

## Learning points

1. CNS histoplasmosis is difficult to diagnose on conventional imaging modalities such as CT or MRI.
2. The literature suggests a possible role for ^18^F-FDG PET/CT scan in the diagnosis of CNS histoplasmosis.
3. CNS histoplasma lesions in HIV-positive patients, similar to toxoplasmosis or tuberculosis, can be hypometabolic on ^18^F-FDG PET/CT scan compared with the grey matter of the brain, suggesting that PET/CT scan likely does not have a role in the diagnostic work-up of these lesions.

## Consent

Informed consent to publish this case (including images and data) was obtained and is held on record.
